# ^18^F-AV-1451 positron emission tomography in Alzheimer’s disease and progressive supranuclear palsy

**DOI:** 10.1093/brain/aww340

**Published:** 2017-01-24

**Authors:** Luca Passamonti, Patricia Vázquez Rodríguez, Young T. Hong, Kieren S. J. Allinson, David Williamson, Robin J. Borchert, Saber Sami, Thomas E. Cope, W. Richard Bevan-Jones, P. Simon Jones, Robert Arnold, Ajenthan Surendranathan, Elijah Mak, Li Su, Tim D. Fryer, Franklin I. Aigbirhio, John T. O’Brien, James B. Rowe

**Affiliations:** 1 1 Department of Clinical Neurosciences, University of Cambridge, Cambridge, UK; 2 2 Istituto di Bioimmagini e Fisiologia Molecolare, Consiglio Nazionale delle Ricerche, Milano, Italy; 3 3 Wolfson Brain Imaging Centre, University of Cambridge, Cambridge, UK; 4 4 Department of Histopathology, Cambridge University Hospital, Cambridge, UK; 5 5 Department of Psychiatry, University of Cambridge, Cambridge, UK; 6 6 Medical Research Council, Cognition and Brain Sciences Unit, Cambridge, UK

**Keywords:** PET, neurodegeneration, tau, Alzheimer’s disease, progressive supranuclear palsy

## Abstract

The ability to assess the distribution and extent of tau pathology in Alzheimer’s disease and progressive supranuclear palsy *in vivo* would help to develop biomarkers for these tauopathies and clinical trials of disease-modifying therapies. New radioligands for positron emission tomography have generated considerable interest, and controversy, in their potential as tau biomarkers. We assessed the radiotracer ^18^F-AV-1451 with positron emission tomography imaging to compare the distribution and intensity of tau pathology in 15 patients with Alzheimer’s pathology (including amyloid-positive mild cognitive impairment), 19 patients with progressive supranuclear palsy, and 13 age- and sex-matched controls. Regional analysis of variance and a support vector machine were used to compare and discriminate the clinical groups, respectively. We also examined the ^18^F-AV-1451 autoradiographic binding in post-mortem tissue from patients with Alzheimer’s disease, progressive supranuclear palsy, and a control case to assess the ^18^F-AV-1451 binding specificity to Alzheimer’s and non-Alzheimer’s tau pathology. There was increased ^18^F-AV-1451 binding in multiple regions in living patients with Alzheimer’s disease and progressive supranuclear palsy relative to controls [main effect of group, *F*(2,41) = 17.5, *P* < 0.0001; region of interest × group interaction, *F*(2,68) = 7.5, *P* < 0.00001]. More specifically, ^18^F-AV-1451 binding was significantly increased in patients with Alzheimer’s disease, relative to patients with progressive supranuclear palsy and with control subjects, in the hippocampus and in occipital, parietal, temporal, and frontal cortices (t’s > 2.2, *P*’s < 0.04). Conversely, in patients with progressive supranuclear palsy, relative to patients with Alzheimer’s disease, ^18^F-AV-1451 binding was elevated in the midbrain (t = 2.1, *P* < 0.04); while patients with progressive supranuclear palsy showed, relative to controls, increased ^18^F-AV-1451 uptake in the putamen, pallidum, thalamus, midbrain, and in the dentate nucleus of the cerebellum (t’s > 2.7, *P*’s < 0.02). The support vector machine assigned patients’ diagnoses with 94% accuracy. The post-mortem autoradiographic data showed that ^18^F-AV-1451 strongly bound to Alzheimer-related tau pathology, but less specifically in progressive supranuclear palsy. ^18^F-AV-1451 binding to the basal ganglia was strong in all groups *in vivo.* Postmortem histochemical staining showed absence of neuromelanin-containing cells in the basal ganglia, indicating that off-target binding to neuromelanin is an insufficient explanation of ^18^F-AV-1451 positron emission tomography data *in vivo*, at least in the basal ganglia. Overall, we confirm the potential of ^18^F-AV-1451 as a heuristic biomarker, but caution is indicated in the neuropathological interpretation of its binding. Off-target binding may contribute to disease profiles of ^18^F-AV-1451 positron emission tomography, especially in primary tauopathies such as progressive supranuclear palsy. We suggest that ^18^F-AV-1451 positron emission tomography is a useful biomarker to assess tau pathology in Alzheimer’s disease and to distinguish it from other tauopathies with distinct clinical and pathological characteristics such as progressive supranuclear palsy.

## Teaser

The extent to which the tau tracer [^18^F]AV-1451 can differentiate between tauopathies is unknown. By comparing patients with Alzheimer’s disease and progressive supranuclear palsy (PSP), Passamonti *et al*. show that [^18^F]AV-1451 displays greater specificity for Alzheimer-related tau pathology than PSP-related pathology. A machine learning algorithm correctly diagnosed 94% of cases.

## Introduction

Alzheimer’s disease and progressive supranuclear palsy (PSP) are both associated with abnormal accumulation of misfolded and aggregated tau protein. In Alzheimer’s disease, oligomeric and aggregated neurofibrillary tau tangles are a major determinant of synaptic/cell dysfunction and death (Goedert *et al.*, 1988; Ballatore *et al.*, 2007; de Calignon *et al.*, 2012), notwithstanding the importance of amyloid-β in its ‘toxic alliance’ with pathological tau (Bloom, 2014). The intensity and distribution of tau in Alzheimer’s disease also correlates with the clinical syndrome and severity and has been considered as one of the primary factors in the neuropathological staging of Alzheimer’s disease (Braak *et al.*, 2006; Murray *et al.*, 2014; Ossenkoppele *et al.*, 2015).

In patients with PSP and in analogous murine models, intra-neuronal and astrocytic aggregates of pathological tau isoforms (in the form of straight filaments) characterize and promote neurodegeneration (Clavaguera *et al.*, 2013). Furthermore, tau pathology is common in other neurological diseases such as fronto-temporal dementia (Hodges *et al.*, 2004), corticobasal degeneration, and may modulate the course of Parkinson’s disease (Spillantini and Goedert, 2001; Irwin *et al.*, 2013), Huntington’s disease (Fernández-Nogales *et al.*, 2014), and multiple sclerosis (Anderson *et al.*, 2008).

Despite the importance of tau pathology in several neurological diseases, it has only recently become possible to assess it using brain imaging in living humans. To be able to measure the burden and distribution of tau pathology in living patients, or those at high risk of developing tau-related disorders, would be a major step forward in the development of disease-modifying therapies targeting the tau protein. Specific markers could also enable pathological characterization of syndromes associated with multiple alternate pathologies, such as frontotemporal dementia and corticobasal degeneration (Alexander *et al.*, 2014). Such biomarkers would ultimately need to be assessed in longitudinal studies and clinical trials, but cross-sectional studies can assess critical properties such as sensitivity to the presence of different diseases and the expected distribution of pathology.

Radioligands have recently been developed for PET to measure *in vivo* binding to aggregated tau, including PBB3 (Maruyama *et al.*, 2013), THK compounds (Okamura *et al.*, 2014), and ^18^F-AV-1451 (Chien *et al.*, 2013; Xia *et al.*, 2013). In autoradiographic studies with post-mortem human brain tissues, the radiotracer ^18^F-AV-1451 co-localizes selectively with hyperphosphorylated tau over amyloid-β plaques (Marquié *et al.*, 2015). In patients with mild cognitive impairment (MCI) and Alzheimer’s disease, there is higher ^18^F-AV-1451 binding in frontal, parietal, and temporal cortices relative to age-matched healthy controls (Okello *et al.*, 2009). Progressively increasing regional ^18^F-AV-1451 binding in Alzheimer’s disease has also been associated with Braak staging of neurofibrillary tau pathology (Schöll *et al.*, 2016; Schwarz *et al.*, 2016), while ^18^F-AV-1451 PET binding patterns mirror the clinical and neuro-anatomical variability in the Alzheimer’s disease spectrum (Ossenkoppele *et al.*, 2016). Specifically, patients with the amnestic presentation of Alzheimer’s disease showed the highest ^18^F-AV-1451 uptake in medial temporal lobe regions including the hippocampus, while patients with the logopenic variant of Alzheimer’s disease displayed increased left hemispheric ^18^F-AV-1451 binding, particularly in posterior temporo-parietal areas implicated in linguistic processes (Ossenkoppele *et al.*, 2016). Performance on domain-specific neuro-psychological tests was also associated with increased ^18^F-AV-1451 uptake in brain regions involved in episodic memory, visuo-spatial skills, and language production or comprehension (Ossenkoppele *et al.*, 2016).

Nevertheless, critical issues remain unresolved, and in particular the value of ^18^F-AV-1451 in differentiating distinct tauopathies as well as the specificity of binding to tau as verified through pathological analyses. Neuropathological data with autoradiography have suggested that the ^18^F-AV-1451 tracer displays strong binding to paired helical filaments characteristic of Alzheimer’s disease (e.g. intra-neuronal and extra-neuronal neurofibrillary tangles and dystrophic neurites), but it does not bind so specifically to the straight tau filaments that are more typical of PSP and non-Alzheimer’s disease tauopathies (e.g. cortico-basal degeneration) (Marquié *et al.*, 2015). However, we have recently found that ^18^F-AV-1451 binds to regions of pathology (i.e. frontal and temporal cortices) in a patient with a *MAPT* gene mutation leading to straight tau filaments and non-Alzheimer's disease dementia (Bevan-Jones, 2016*b*). It has also been proposed that the ^18^F-AV-1451 tracer displays off-target binding, specifically to neuromelanin-containing cells. This was supported by evidence in patients with Parkinson’s disease, *in vivo*, in the midbrain; and post-mortem, in retinal and brain tissues in porcine and rodent models (Hansen *et al.*, 2016).

In this study, we sought to evaluate the utility of ^18^F-AV-1451 PET imaging in Alzheimer’s disease versus non-Alzheimer’s disease tauopathies. We used dynamic PET imaging with kinetic modelling, rather than standardized uptake value ratios (SUVR), in part to accommodate variations in cerebral perfusion that can reduce reliability of SUVR. We aimed to identify the patterns of ^18^F-AV-1451 uptake in patients with Alzheimer’s disease and contrast these patterns with those that were expected in patients with PSP on the basis of previous studies (Johnson *et al.*, 2016; Ossenkoppele *et al.*, 2016; Schöll *et al.*, 2016; Schwarz *et al.*, 2016).

The value of comparing these two clinical groups lies not in their differential diagnosis, which is clear on clinical grounds alone, but in testing the ligand’s binding against well-established clinico-pathological correlations and distinct distributions of tau pathology. These two disorders represent different kinds of tauopathy, with paired helical versus straight filamentous tau. Evidence on ^18^F-AV-1451’s binding distributions, its off-target binding, and clinical correlations would directly inform the design of forthcoming clinical trials of anti-tau therapies in these diseases.

Overall, we aimed to: (i) identify the patterns of ^18^F-AV-1451 binding in patients with Alzheimer’s disease, relative to patients with PSP as well as sex- and age-matched healthy controls; (ii) test whether ^18^F-AV-1451 binding was associated with disease severity in Alzheimer’s disease and PSP; and (iii) assess whether regional ^18^F-AV-1451 binding could distinguish between Alzheimer’s disease and PSP groups. We combined patients with clinical diagnostic criteria for Alzheimer’s disease and MCI patients who had biomarker evidence of Alzheimer’s disease pathology (i.e. with a positive scan for amyloid), based on the fact that these two groups represent a continuum of disease (Okello *et al.*, 2009). In view of the suggested effect of off-target binding, we also examined ^18^F-AV-1451 uptake in relation to AT8 immunohistochemistry of hyperphosphorylated tau protein and tinctorial stain for neuromelanin, in post-mortem sections from a patient with Alzheimer’s disease, PSP, and a similarly aged control from the Cambridge Brain Bank.

Our principal hypotheses were that: (i) patients with Alzheimer’s disease and those with MCI and PET scans positive for amyloid would show increased ^18^F-AV-1451 binding in the cortical and subcortical areas associated with Alzheimer’s pathology, including the medial temporal lobe as well as frontal, parietal, and temporal cortices (Serrano-Pozo *et al.*, 2011); (ii) patients with PSP would display increased ^18^F-AV-1451 binding especially in the midbrain and basal ganglia, with likely additional binding in frontal cortex (including the motor areas) and supramarginal gyrus, a set of subcortical and cortical regions that have been shown to display tau pathology in PSP (Schofield *et al.*, 2005; Dickson *et al.*, 2007; Smith *et al.*, 2016); and (iii) PSP and Alzheimer’s disease patients would be distinguishable based on the regional ^18^F-AV-1451 binding levels, particularly in the hippocampus and midbrain, two key subcortical regions that show highly distinct neuropathological changes in Alzheimer’s disease and PSP, respectively.

## Materials and methods

### Participants

The current study was conducted within the context of the Neuroimaging of Inflammation in MemoRy and Other Disorders (NIMROD) project (Bevan-Jones *et al.*, 2016*a*). We recruited 19 patients with PSP [‘probable PSP’ by Movement Disorder Society criteria (Litvan *et al.*, 1996*a*, *b*), representing the ‘classical phenotype’, which is sometimes referred to as Richardson’s syndrome], nine patients meeting diagnostic criteria for probable Alzheimer’s disease (McKhann *et al.*, 2011), and six patients with MCI and biomarker evidence of Alzheimer’s disease (i.e. amyloid pathology). MCI was defined as a Mini-Mental Score Examination (MMSE) >24 with a memory impairment at least 1.5 standard deviations (SD) below that expected for age and education (Petersen *et al.*, 1999). All participants with MCI had a positive Pittsburgh compound B (PiB) PET scan (assessing *in vivo* amyloid pathology). Thirteen age- and sex-matched healthy controls with no history of major psychiatric or neurological illnesses, head injury or any other significant medical co-morbidity were also included to allow group-wise comparisons with the clinical cohorts. All participants were aged over 50 years, had sufficient proficiency in English for cognitive testing and had no contraindications to MRI. Patients and healthy controls were identified from the specialist clinics for memory disorders and PSP at the Cambridge University Hospitals NHS Trust and from registers held by the Dementias and Neurodegenerative Diseases Research Network (DeNDRoN); part of the NIHR Clinical Research Network. All participants had full mental capacity and provided written informed consent which was approved by the local ethical committee, in accord to the Declaration of Helsinki. As some of the assessment scales required carers’ input for completion, we also obtained informed written consent from contributory carers.

### Clinical and neuroimaging assessment

#### Clinical and cognitive assessment

Participants underwent an initial assessment that included clinical indices of disease severity (e.g. Progressive Supranuclear Palsy Rating Scale) (Golbe and Ohman-Strickland, 2007), demographic measures, and neuropsychological tests [MMSE and Addenbrooke’s Cognitive Examination-Revised (ACE-R)].

#### MRI data acquisition and preprocessing

Participants underwent an MRI session acquired on a 3 T scanner (Siemens Magnetom Tim Trio and Verio scanner; www.medical.siemens.com) using a MP-RAGE T_1_-weighted sequence (all groups). The T_1_-weighted sequence (repetition time = 2300 ms, echo time = 2.98 ms, field of view = 240 × 256mm^2^, 176 slices of 1 mm thickness, flip angle = 9°) was used to facilitate tissue class segmentation (grey and white matter, together with CSF), and to allow non-rigid registration of standard space regions of interest (using a modified version of the Hammers atlas that included the midbrain and dentate nucleus of the cerebellum) (Hammers *et al.*, 2003) to subject MRI space. Each T_1_ image was non-rigidly registered to the ICBM2009a template brain using ANTS (http://www.picsl.upenn.edu/ANTS/) and the inverse transform was applied to the Hammers atlas (resliced from MNI152 to ICBM2009a space) to bring the regions of interest to subject MRI space.

### PET data acquisition and preprocessing

All participants (i.e. patients with Alzheimer’s disease, with MCI, with PSP, and control subjects) underwent ^18^F-AV-1451 PET imaging to assess the extent and intensity of brain tau pathology. Subjects with MCI also underwent ^11^C-PiB PET imaging to assess the density of amyloid-β deposits as an indication of Alzheimer’s disease amyloid pathology. All radioligands were prepared at the Wolfson Brain Imaging Centre (WBIC), University of Cambridge, with high radiochemical purity (>95%). ^11^C-PiB was produced with specific activity of >150 GBq/μmol, while ^18^F-AV-1451 specific activity was of 216 ± 60 GBq/μmol at the end of synthesis. PET scanning was performed on a GE Advance PET scanner (GE Healthcare) and a GE Discovery 690 PET/CT. A 15-min ^68^Ge/^68^Ga transmission scan was used for attenuation correction on the Advance, which was replaced by a low dose CT scan on the Discovery 690. The emission protocols were the same on both scanners: 550 MBq ^11^C-PiB injection followed by imaging from 40–70 min post-injection and 90 min dynamic imaging (58 frames) following a 370 MBq ^18^F-AV-1451 injection.

Each emission frame was reconstructed using the PROMIS 3D filtered back projection algorithm into a 128 × 128 matrix 30 cm transaxial field of view, with a transaxial Hann filter cut-off at the Nyquist frequency (Kinahan and Rogers, 1989). Corrections were applied for randoms, dead time, normalization, scatter, attenuation, and sensitivity. Each emission image series was aligned using SPM8 to correct for patient motion during data acquisition (www.fil.ion.ucl.ac.uk/spm/software/spm8).

The mean aligned PET image, and hence the corresponding aligned dynamic PET image series, was rigidly registered to the T_1_-weighted image using SPM8 to extract values from both the Hammers atlas regions of interest and those in a reference tissue defined in the superior grey matter of the cerebellum using a 90% grey matter threshold on the grey matter probability map produced by SPM8 smoothed to PET resolution. The superior cerebellum was used as reference region as it is considered to have little or no tau pathology in either PSP or Alzheimer’s disease (Williams *et al.*, 2007; Okello *et al.*, 2009; Dickson, 2010; Schöll *et al.*, 2016; Schwarz *et al.*, 2016). This was confirmed in our post-mortem cases (Supplementary material). All region of interest data, including the reference tissue values, were corrected for CSF partial volumes through division with the mean region of interest probability (normalized to 1) of grey plus white matter segments, each smoothed to PET resolution. To test whether correction for CSF affected the main results, we repeated all the ^18^F-AV-1451 PET analyses using data not corrected for CSF (see ‘PET statistical analyses’ and ‘Results’ sections).

^11^C-PiB data were quantified using SUVR by dividing the mean CSF-corrected radioactivity concentration in each region of interest by the corresponding mean radioactivity concentration in the reference tissue region of interest (whole cerebellum). For ^18^F-AV-1451 non-displaceable binding potential (BP_ND_), a measure of specific binding, was determined for each Hammers atlas region of interest using a basis function implementation of the simplified reference tissue model (SRTM) operating upon the Hammers atlas and reference tissue region of interest data, both with and without CSF correction (Gunn *et al.*, 1997). ^11^C-PiB data were treated as dichotomous measures (i.e. positive or negative) and considered positive if the ratio of the average SUVR values across the cortical and cerebellar regions of interest was >1.5, as previously described (Hatashita and Yamasaki, 2013).

### PET statistical analyses

To compare ^18^F-AV-1451 binding across groups (Alzheimer’s disease/MCI PiB+, PSP, and controls), individual region of interest BP_ND_ values for ^18^F-AV-1451 were used in a repeated-measures general linear model (GLM) to test for the main effect of region of interest, main effect of group, and group × region of interest interaction. Age and education were included as covariates of no interest. For the Alzheimer’s disease/MCI PiB+ and PSP groups, we tested for correlations between regional ^18^F-AV-1451 BP_ND_ and disease severity using the ACE-R scores for Alzheimer’s disease/MCI PiB+ patients and the Progressive Supranuclear Palsy Rating Scale for PSP patients with Pearson’s correlation (with partial correlations accounting for age and education). All analyses were repeated using ^18^F-AV-1451 BP_ND_ values that were not corrected for CSF partial volume effects. To assess the ability of ^18^F-AV-1451 BP_ND_ to distinguish Alzheimer’s disease patients from PSP cases, subject-specific ^18^F-AV-1451 data in a set of regions of interest were input as key features in a support vector machine (SVM), a multivariate supervised statistical learning method suitable for neuroimaging modalities (Cortes and Vapnik, 1995). A reduced group of regions of interest considered as the most characteristic regions of interest affected by tau pathology in Alzheimer’s disease and PSP was selected (i.e. superior/inferior temporal cortex, lateral occipital cortex, inferior parietal cortex, and hippocampus for Alzheimer’s disease, and basal ganglia and midbrain for PSP); noting that the regions of interest included in the SVM were identical for both groups. This extended the whole-brain hierarchical cluster analysis described in the Supplementary material. The accuracy of each region of interest to discriminate between the Alzheimer’s disease and PSP groups was computed using an SVM classifier with a K means cross-validation (K = 5) scheme with a linear kernel and standard cost parameter of 1.

### Neuropathological methods

#### Tissue samples preparation

Post mortem brain tissue from three subjects (one Alzheimer’s disease case, one PSP patient, and one healthy control with similar age) from the Cambridge Brain Bank was included in this study. The autoradiographic and immunohistochemical analyses were conducted in different cases from those included in the PET *in vivo* study. Tissue collection was approved by the local institutional review board. Neuropathological diagnoses were performed according to standardized protocols, on 15 blocked regions of cortex and subcortical regions. For this study, additional blocks of frozen brain tissue were obtained from the anterior hippocampus, midbrain, basal ganglia, and frontal cortex. Sections (20-μm thick) were cut in a cryostat (Leica CM30505 S Research Cryostat) mounted on Thermo Scientific superfrost plus slides and used for ^18^F-AV-1451 phosphor screen, phosphorylated-tau immunoreactivity (AT8), and tinctorial stain for neuromelanin (Masson-Hamperl stain).

#### ^18^F-AV-1451 phosphor screen autoradiography

^18^F-AV-1451 for autoradiographic studies was synthesized in the same way as described above. ^18^F-AV-1451 phosphor screen autoradiography was performed following a previously published protocol by Marquiè and collaborators (2015). In brief, 20 μm-thick frozen brain sections were fixed in 100% methanol at room temperature for 20 min and then transferred to a bath containing high specific activity ^18^F-AV-1451 in 10 mM phosphate-buffered saline (PBS) with a radioactivity concentration of ∼20 μCi/ml. Adjacent brain slices were placed in a bath that was identical in all aspects except that unlabelled AV-1451 was added to yield 1 μM chemical concentration, a blocking condition sufficient to saturate essentially all available specific binding sites of tau. After incubation for 60 min, racks of slides were removed from the respective radioactive solutions and briefly incubated in a series of wash baths to remove unbound radiotracer. Wash solutions and incubation times were: 10 mM PBS for 1 min, 70% ethanol/30% PBS for 2 min, 30% ethanol/70% PBS for 1 min, and lastly 100% 10 mM PBS for 1 min. Racks were removed from the final wash solution, and slides were allowed to air dry before transfer to a storage phosphor screen (GE healthcare) that had been photobleached immediately before by exposure on a white light box for a minimum of 15 min. The slides and phosphor screen were enclosed in an aluminium film cassette and set away from sources of radioactivity for the duration of the overnight exposure period. The cassette was opened and the slides were removed from the exposed screen, which was mounted on the digital imaging system (CR 35 BIO, Durr medical). Scanning of screens was controlled by Aida Image Analyser v.4.27 using 600 dpi resolution (∼42 μm sampling interval). Digital images were saved at full resolution and pixel depth. Images from adjacent brain slices incubated in the unblocked (high specific activity ^18^F-AV-1451 only) and blocking (^18^F-AV-1451 plus 1 μm unlabelled AV-1451) conditions were compared to estimate total and non-specific binding of ^18^F-AV-1451.

## Results

### Demographics and cognitive variables of patients in the PET *in vivo* study

There were no statistically significant differences between patient and control groups in terms of age or sex (Table 1). Shorter education was reported by patients with PSP relative to other groups (Table 1). One interpretation of this difference is that control cohorts over-represent people from higher socio-economic groups and with longer education; however, low education and its effects on health may also be a risk factor for the development of PSP (Litvan *et al.*, 2016). Age and education were included as covariates of no interest in the statistical models assessing the main effect of region, main effect of group, and group × region of interest interactions. As expected, there was a significant main effect of group for cognitive measures, driven by reduced MMSE and ACE-R scores in Alzheimer’s disease/MCI+ and PSP patients relative to healthy controls (Table 1).

**Table 1:** Participant details and group differences by one-way ANOVA or chi-squared test

	**AD/MCI+ (*n* = 15)**	**PSP (*n* = 19)**	**Healthy controls (*n* = 13)**	**Group difference**
Sex (male/female)	9/6	11/8	6/7	N/S
Age, years (SD, range)	71.6 (±8.7, 54–85)	69.5 (±5.8, 52–79)	67.2 (±7.3, 55–80)	*F* = 1.2, *P* = 0.3
Education, years (SD, range)	14.3 (±3.3, 10–19)	11.9 (±1.8, 10–17)	15.8 (±1.9, 11–19)	*F* = 10.2, *P* = 0.0003
MMSE (SD, range)	25.5 (±2.8, 18–28)	26.1 (±4.5, 13–30)	29.3 (±0.7, 28–30)	*F* = 4.9, *P* = 0.012
ACE-R (SD, range)	75.9 (±11.0, 51–89)	78.7 (±15.8, 36–95)	95.5 (±3.0, 89–99)	*F* = 10.3, *P* = 0.0002
PSP Rating Scale (SD, range)	–	43.6 (±15.8, 15–74)	–	–

Values are mean (±SD, range).

AD/MCI+ = Alzheimer’s disease/mild cognitive impairment (amyloid-positive from PiB-PET scan); MMSE = Mini-Mental State Examination; ACE-R = Addenbrookes’ Cognitive Examination, Revised. N/S = not significant at *P* < 0.05 (uncorrected).

### ^18^F-AV-1451 binding in relation to clinical diagnosis

**Figure 1:**
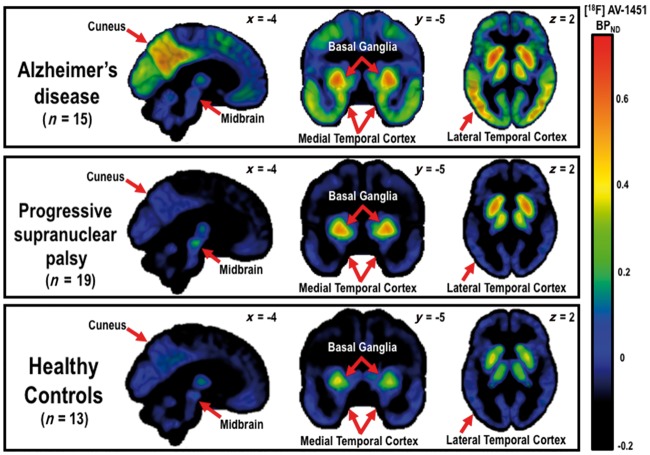
**BP_ND_ for ^18^F-AV-1451 for Alzheimer’s disease, including PiB positive MCI, PSP, and healthy controls.** Note the ^18^F-AV-1451 binding in the basal ganglia in all groups, albeit higher in Alzheimer’s disease and PSP patients. Patients with Alzheimer’s disease also showed increased ^18^F-AV-1451 binding in medial temporal lobe regions and widespread neocortical areas, relative to controls and PSP patients, while PSP patients had increased high ^18^F-AV-1451 binding to the midbrain, relative to patients with Alzheimer’s disease and control subjects (see Fig. 2 and ‘Results’ section in the main text for quantitative analyses).

**Figure 2:**
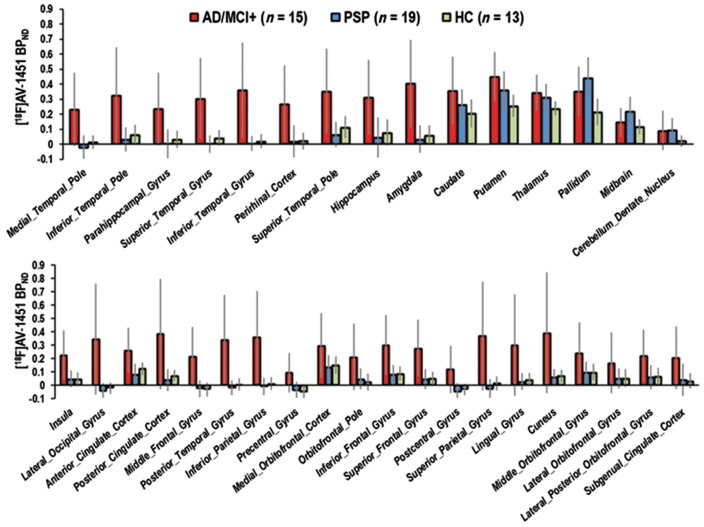
**Mean (±SD) ^18^F-AV-1451 BP_ND_ in each region of interest for the participant groups: Alzheimer’s disease and amyloid-positive MCI; PSP, and healthy controls.** The ^18^F-AV-1451 BP_ND_ data reported here are corrected for CSF volume. See the ‘Results’ section for statistics related to CSF corrected and uncorrected data. AD = Alzheimer’s disease; MCI+ = amyloid-positive MCI; HC = healthy control.

The mean ^18^F-AV-1451 BP_ND_ PET map in each group (Fig. 1) and quantitative region of interest analyses (Fig. 2), indicated high ^18^F-AV-1451 binding in the basal ganglia in all groups including controls. In the repeated-measures ANOVA of regional binding, we found a significant main effect of group [*F*(2,41) = 17.5, *P* = 0.00001] and a region of interest×group interaction [*F*(2,68) = 7.5, *P* < 0.00001], although there was no main effect of regions of interest [*F*(2,34) = 0.8, *P* = 0.8] (Fig. 2). The group and interaction effects were driven in part by greater ^18^F-AV-1451 BP_ND_ in the Alzheimer’s disease/MCI+ group relative to the PSP and control groups, in cortical and subcortical areas including frontal, parietal, lateral temporal, and occipital cortices as well as the hippocampus and other medial temporal lobe regions (*post hoc t*-tests, t’s > 2.2, *P*’s < 0.04) (Fig. 2). The PSP group, relative to the Alzheimer’s disease group, showed increased ^18^F-AV-1451 BP_ND_ in the midbrain (t = 2.1, *P* < 0.04); while, relative to controls, PSP patients showed increased ^18^F-AV-1451 BP_ND_ in the putamen, pallidum, thalamus, midbrain, and dentate nucleus of the cerebellum (t’s > 2.7, *P* < 0.02) (Fig. 2).

Repeating the analyses using ^18^F-AV-1451 BP_ND_ values that were not corrected for CSF partial volume effects yielded similar results [F(2,36) = 1.1, *P* = 0.2, for the main effect of regions of interest; *F*(2,41) = 16.7, *P* < 0.00001 for the main effect of group; and F(2,72) = 6.3, *P* < 0.00001 for the group × region of interest interaction]. We then tested whether regional ^18^F-AV-1451 BP_ND_ related to disease severity. In the Alzheimer’s disease/MCI+ group, there was no significant correlation between ACE-R score and ^18^F-AV-1451 BP_ND_ in any region of interest (*P*’s > 0.14). Similarly, in the PSP group, we found no significant correlation between ^18^F-AV-1451 BP_ND_ in any region of interest and disease severity, as assessed via the Progressive Supranuclear Palsy Rating Scale (*P*’s > 0.16). Repeating the correlation analyses when using the ^18^F-AV-1451 BP_ND_ values that were not corrected for CSF volume yielded similar non-significant results (*P*’s > 0.1).

### Classification of cases by ^18^F-AV-1451 BP_ND_

**Figure 3:**
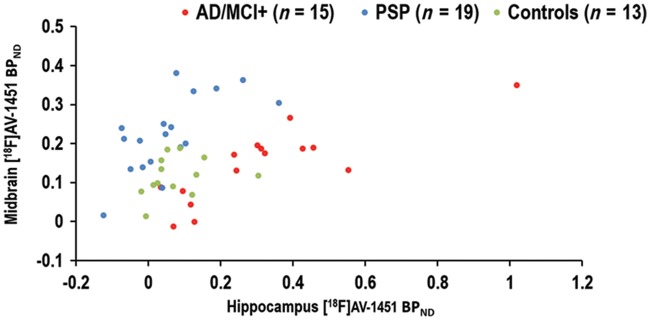
**Individual ^18^F-AV-1451 BP_ND_ values in the hippocampus (*x*-axis) and midbrain (*y*-axis) in patients with Alzheimer’s disease (AD) and amyloid-positive MCI (MCI+; red dots), PSP (cyan dots), and healthy control subjects (green dots).** Note the clear bivariate separation of AD/MCI+ from PSP patients.

The SVM analysis using ^18^F-AV-1451 BP_ND_ values in a subset of regions of interest was able to separate the Alzheimer’s disease/MCI+ patients from PSP cases with a classification accuracy of 94.1%. The accuracy for the other pair-wise comparisons is as follows: Alzheimer’s disease/MCI+ versus controls = 85.7%; PSP versus controls = 90.6% (Fig. 3 for data plot from two characteristic regions of interest). In the Supplementary material we also report the accuracy of pair-wise comparisons between groups using hierarchical cluster analyses, based on the regional distribution of ^18^F-AV-1451 BP_ND_ across the whole brain (Bevan Jones *et al.*, 2016*a*).

### Phosphor screen autoradiography and immunohistochemistry post-mortem

**Figure 4:**
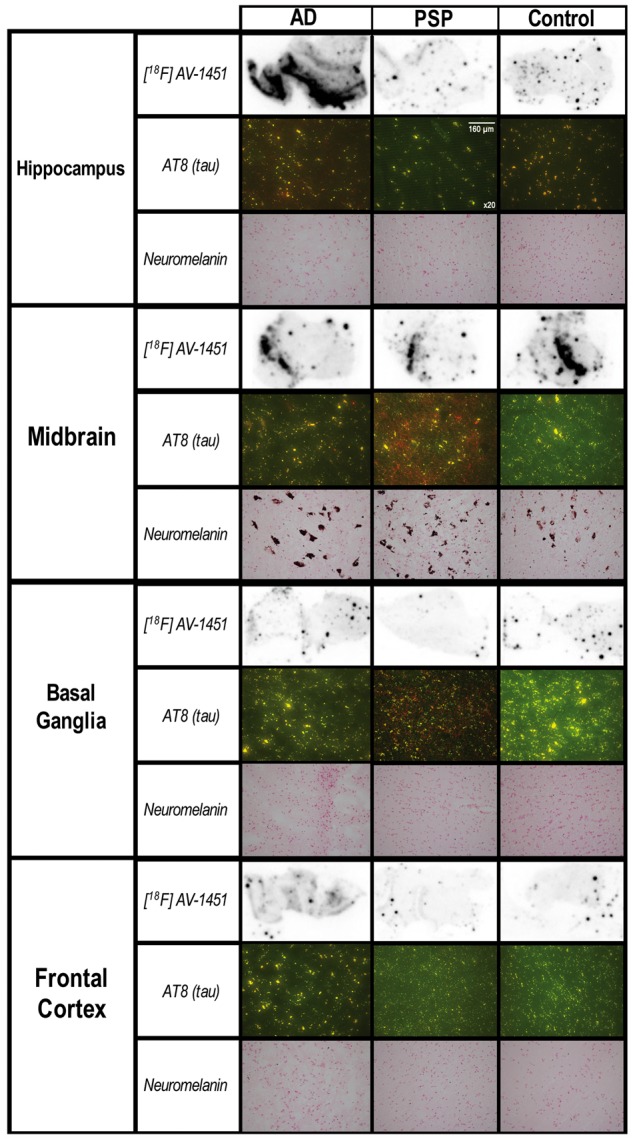
**Post-mortem data.** The figure aligns the ^18^F-AV-1451 autoradiographic binding in key regions of interest in an Alzheimer’s disease (AD) case, a patient with PSP, and a control of similar age. Immunohistochemistry data assessing hyperphosphorylated tau (AT8, red), and neuromelanin staining (dark brown) are also shown for the same cases and regions of interest. There is ^18^F-AV-1451 binding in the hippocampus and, to a lesser extent, in the frontal cortex in Alzheimer’s disease. In contrast, ^18^F-AV-1451 binding to the midbrain slices was not specific to PSP but was also detected in the Alzheimer’s disease and control cases, who showed little or no tau pathology in the midbrain. Despite the *in vivo*^18^F-AV-1451 binding to the basal ganglia in all groups (including controls, see Figs 1 and 2), post-mortem ^18^F-AV-1451 binding to the basal ganglia was sparse and non-specific in these three cases. Note the absence of neuromelanin-containing cells in the basal ganglia and cortical regions. The magnification of the immunohistochemistry pictures (AT8) and neuromelanin staining is ×20.

A summary of the autoradiography results, AT8 immunohistochemistry data, and neuromelanin staining in post-mortem Alzheimer’s disease, PSP, and control case is shown in Fig. 4.

The autoradiography phosphor screen analyses in the Alzheimer’s disease brain tissue sample revealed that the hippocampus had the highest and most specific binding of the ^18^F-AV-1451 radiotracer. ^18^F-AV-1451 binding was also found in the frontal cortex in the Alzheimer’s disease case, although to a lesser extent than in the hippocampal slice. In contrast, sparse and non-specific ^18^F-AV-1451 binding was found in the Alzheimer’s disease basal ganglia tissue.

The PSP and control tissues showed overall sparse and non-specific ^18^F-AV-1451 binding, including hippocampus, midbrain, basal ganglia, and frontal cortex.

Abundant hyperphosphorylated tau protein was detected in the hippocampus of the Alzheimer’s disease case, while small and punctate tau staining was found in the midbrain and frontal cortex of the same patient, which is overall consistent with the results of the phosphor screen autoradiography. Although hyperphosphorylated tau protein was found in the frontal cortex in the Alzheimer’s disease case, its relatively low density could be due to a slow cortical disease progression in this particular patient.

The PSP tissue displayed high concentration of hyperphosphorylated tau in the midbrain and basal ganglia, while the Alzheimer’s disease brain displayed little AT8 staining in the basal ganglia.

As expected, the control brain did not show AT8 immunoreactivity in any of the regions of interest examined.

Neuromelanin-containing cells were only observed in the midbrain in all post-mortem cases. Of note, no neuromelanin-containing cells were found in the basal ganglia in either the Alzheimer’s disease, PSP or control case, which is in contrast to the strong *in vivo*^18^F-AV-1451 binding of this radiotracer to the same region of interest.

## Discussion

The principal result of our study is that PET imaging with the radiotracer ^18^F-AV-1451 revealed distinct patterns of binding in Alzheimer’s disease and its prodromal state of MCI, in comparison to the primary tauopathy of PSP. The relatively large size of our PET study confirmed the high accuracy of discrimination between the clinical groups using ^18^F-AV-1451 BP_ND_ data, with a simple support vector machine and indeed by visual inspection (Figs 1, 2 and 3). However, despite this heuristic potential of ^18^F-AV-1451 as a tau biomarker, caution in the interpretation of its binding targets is indicated by the neuropathological and autoradiographic data (Marquiè *et al.*, 2015). In particular, while ^18^F-AV-1451 strongly bound to Alzheimer’s disease-related tau pathology, non-specific binding of the same tracer can be found in patients with PSP and control subjects (Marquiè *et al.*, 2015). Nevertheless, our post-mortem data suggest that off-target binding to neuromelanin is not a sufficient explanation of the BP_ND_ for ^18^F-AV-1451, at least in the context of PSP, and in some critical regions as the basal ganglia. For instance, we found *in vivo* significant ^18^F-AV-1451 binding in the basal ganglia (in all groups including controls) in the absence of post-mortem neuromelanin-containing cells. This indicates that neuromelanin is not the principal target of off-target binding for ^18^F-AV-1451, but there may be other off-target binding sites that have as yet not been identified, including non-tau targets in disorders associated with predominantly TDP-43 pathology (Bevan-Jones *et al.*, 2016*c*).

For ^18^F-AV-1451 PET to meet its full potential as a biomarker to stratify or monitor the effect of disease-modifying drugs in future clinical trials, additional properties would therefore need to be established. In particular, further work is needed to demonstrate changes in ^18^F-AV-1451 PET over time, or in response to treatment. A cross-sectional study as this one cannot be used to infer longitudinal changes, but it can be employed to inform and model a biomarker’s potential. More specifically, the relevance of ^18^F-AV-1451 is increased by the demonstration that its binding patterns recapitulate *in vivo* the established post-mortem distributions of tau pathology in Alzheimer’s disease and PSP. In addition, ^18^F-AV-1451 PET may have biomarker potential for the differential diagnosis of equivocal cases: while the distinction between Alzheimer’s disease and PSP can be readily made on clinical grounds, patients with PSP-parkinsonism clinically resemble Parkinson’s disease (Williams *et al.*, 2005).

In contrast to previous results (Johnson *et al.*, 2016; Ossenkoppele *et al.*, 2016), ^18^F-AV-1451 binding was not correlated with disease severity in our groups (i.e. severity of cognitive impairment in patients with Alzheimer’s disease and Progressive Supranuclear Palsy Rating Scale in patients with PSP). There are several possible explanations for the lack of a correlation in our study, including lack of statistical power (type II error) or the use of clinical measures that were not sufficiently sensitive to describe the full spectrum of clinical variability in Alzheimer’s disease and PSP. Alternatively, it may be that ^18^F-AV-1451 binding is inherently limited in staging disease severity in Alzheimer’s disease and PSP, analogous to the PiB tracer in Alzheimer’s disease (Hatashita and Yamasaki, 2010).

Technical considerations in assessing the ^18^F-AV-1451 binding post-mortem and in estimating BP_ND_*in vivo* must also be discussed. First, it is possible that in the autoradiographic protocol (Marquié *et al.*, 2015), ethanol washing, and other procedures may have affected the labelling with ^18^F-AV-1451, especially in the basal ganglia. Second, our PET analyses employed correction of ‘partial volume effects’, resulting from CSF volume within each region. This mitigates the potential influence of brain volume loss seen in Alzheimer’s disease and PSP. Nevertheless, using uncorrected PET data yielded qualitatively similar results in terms of the main effect of group and group × region of interest interaction, which suggests that we avoided ‘over-correcting’ the BP_ND_ values based on cortical and subcortical atrophy, and the consequent inferential error from CSF volume and its correction.

Interestingly, the regions with the most significant group differences in ^18^F-AV-1451 BP_ND_ in Alzheimer’s disease and PSP *in vivo* were those predicted from prior post-mortem studies for each disease. More specifically, the clinical syndromes of Alzheimer’s disease and biomarker positive MCI were associated with increased ^18^F-AV-1451 BP_ND_ in widely distributed sub-cortical and cortical areas that have been consistently implicated in the pathogenesis and progression of Alzheimer’s disease (e.g. hippocampus, amygdala as well as frontal, parietal, temporal, and occipital cortices) (Braak and Braak, 1995). Conversely, PSP was associated with a pattern of increased ^18^F-AV-1451 BP_ND_ in the basal ganglia, midbrain, and dentate nucleus of the cerebellum, consistent with the pathophysiology of the disease (Hauw *et al.*, 1994; Litvan *et al.*, 1996*a*, *b*). Together, these data demonstrated that the ^18^F-AV-1451 ligand recapitulates *in vivo* the typical neuropathological changes seen in Alzheimer’s disease and PSP, although it cannot be assumed that the cellular and/or molecular targets of ^18^F-AV-1451 binding are the same in both disorders.

^18^F-AV-1451 BP_ND_ in selected regions of interest also distinguished Alzheimer’s disease cases from PSP patients with an accuracy of 94% which suggests the potential of this radio-tracer to discriminate *in vivo* among different tauopathies. The value of this analysis is obviously not as a diagnostic biomarker, as clinical features readily distinguish the groups, but rather represents an early step in the process of validating ^18^F-AV-1451 PET as a biomarker for tauopathies. Multicentre replication with larger samples and broader diagnostic spectra are necessary, including for example, patients with frontotemporal dementia, corticobasal syndrome, or presymptomatic individuals with high risk of developing tau-related neurodegenerative disorders (e.g. carrying specific gene mutations).

Finally, we note that our data are specific to ^18^F-AV-1451, and do not necessarily generalize to other radioligands. Further work is required to determine the specificity of ^18^F-AV-1451 and other candidate ligands’ binding to the different isoforms of tau protein, their differential modes of modification (e.g. phosphorylation, acetylation) and aggregation (e.g. oligomeric states or neurofibrillar tangles). These issues are of high relevance for this and other studies because: (i) Alzheimer’s disease is characterized by balanced 3R/4R isoforms, while PSP pathology is mainly a 4R isoform tauopathy (Buée and Delacourte, 1999; Espinoza *et al.*, 2008); and (ii) the toxicity of tau aggregates may be driven by oligomers rather than tangles.

In conclusion, we suggest that ^18^F-AV-1451 is a useful PET ligand for *in vivo* studies in clinical populations with Alzheimer’s disease pathology and non-Alzheimer’s disease primary tauopathies such as PSP, despite the potential contribution of non-specific or ‘off-target’ binding. The brain regions with increased ^18^F-AV-1451 binding were those predicted from the well-established patterns of neurodegeneration in both diseases, and are in keeping with the cognitive and motor features classically seen in Alzheimer’s disease and PSP clinical syndromes, respectively. Together, our current findings support the further use of ^18^F-AV-1451 PET *in vivo* and *in vitro* to evaluate tau pathology in studies of dementia and neurodegeneration.

## Supplementary Material

Supplementary Data

## Abbreviations

BP_ND_: non-displaceable binding potential
MCI: mild cognitive impairment
PiB: Pittsburgh compound B
PSP: progressive supranuclear palsy
