# Supplementary material for: 18F-AV-1451 positron emission tomography in Alzheimer’s disease and progressive supranuclear palsy
Source: Brain. 2017 Jan 24;140(3):781–91. doi: 10.1093/brain/aww340 (PMC5382948; doi:10.1093/brain/aww340)
Supplement: Supplementary Data [file aww340_supp.pdf]

Hierarchical cluster analyses

To test whether the distribution of [^{18}F]AV-1451 BP_{ND} differed across groups, we initially performed hierarchical clustering of individuals according to the Pearson correlation of their region-wise [^{18}F]AV-1451 binding across the whole-brain, collapsed across hemispheres (see Bevan-Jones et al., 2016 for further details of this method). The similarities driving this classification can be visualised using multi-dimensional scaling (**Supplementary Figure 1**).

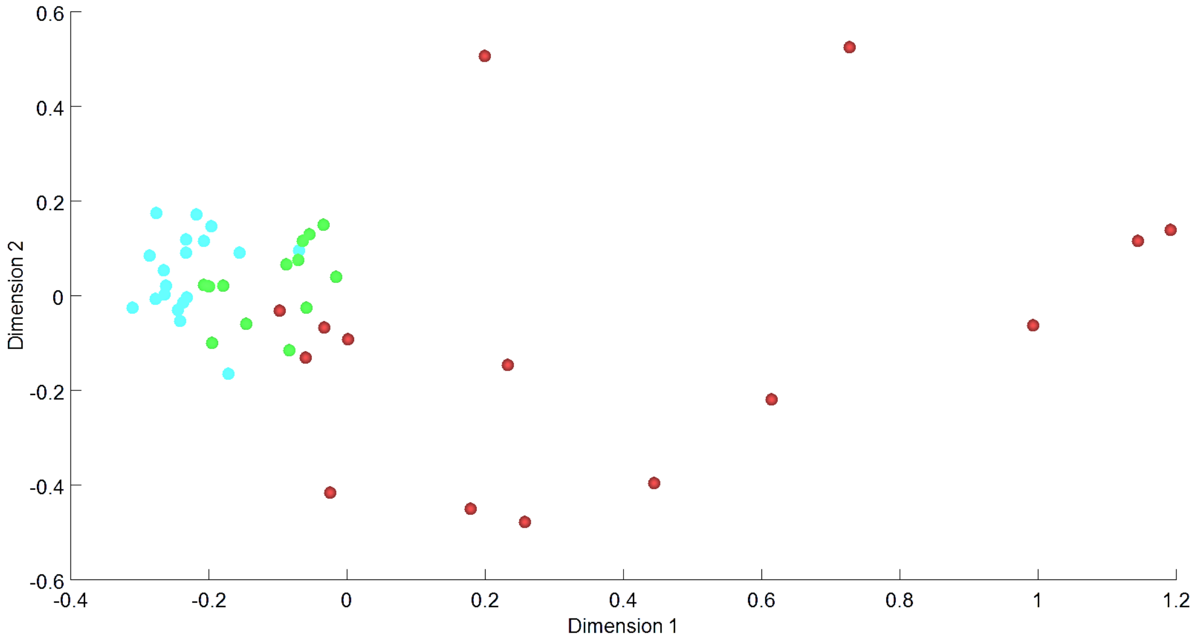

Supplementary Figure 1. Two dimensional scaling of [^{18}F]AV-1451 BP_{ND} distribution across groups (**green**, healthy controls; **cyan**, progressive supranuclear palsy patients (PSP); **red**, Alzheimer's disease/mild cognitive amyloid PiB+ patients (AD/MCI+)). The fitting method was minimisation of squared metric stress.

The accuracy for pair-wise comparisons using hierarchical cluster analyses was as follows: PSP vs. healthy controls=84.4%; AD/MCI+ vs. PSP=88.2%; AD/MCI+ vs. healthy controls=82%.

The hierarchical cluster approach compares groups across the whole-brain, but there were *a priori* reasons to believe that distributional differences would be stronger in certain regions; for example, tau is known to preferentially accumulate in the hippocampus and parietal lobes in AD (Serrano-Pozo et al., 2011) and midbrain in PSP (Dickson et al., 2007). We therefore applied a support vector machine to the regional data, to assess whether classification accuracy could be improved and explore whether the brain regions

characteristic for each clinical group were the strongest drivers of group dissociation (see main text).

Tau immune-histochemistry (AT8) in the superior cerebellum

Only very sparse and punctate tau staining was found in the superior cerebellum of the PSP case (**Supplementary Figure 2A**), while no tau pathology was seen in the AD and control case (see respectively **Supplementary Figure 2B and C)**. This is overall consistent with the notion that the superior cerebellum displays little or no tauopathy compared to other brain areas in either PSP and AD (NINDS PSP consensus criteria, William 2007; Dickson 2010; Okello et al., 2009; Schöll et al., 2016; Schwarz et al., 2016). These findings also corroborated the use of the superior cerebellum as reference regions in the PET analyses (see main text).

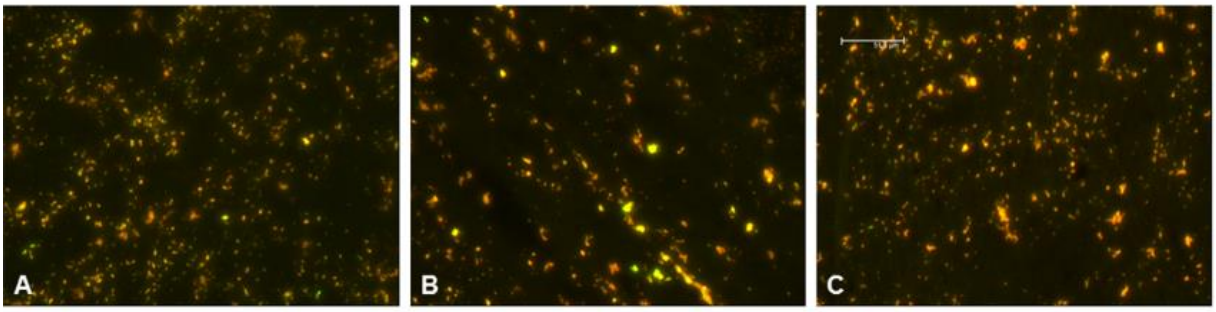

Supplementary Figure 2. *Post mortem* immune-histochemistry data assessing hyper-phosphorylated tau (AT8, red) in the superior cerebellum in a case with progressive supranuclear palsy (**A**), Alzheimer's disease (**B**), and no neurodegenerative disorders (healthy control) (**C**). Note the small and punctate tau staining in the superior cerebellum of the PSP case and the absence of tau pathology in the other cases. The magnification of the immunohistochemistry pictures is x20.
